# Subamolide A Induces Mitotic Catastrophe Accompanied by Apoptosis in Human Lung Cancer Cells

**DOI:** 10.1155/2013/828143

**Published:** 2013-02-24

**Authors:** Jen-Yu Hung, Ching-Wen Wen, Ya-Ling Hsu, En-Shyh Lin, Ming-Shyan Huang, Chung-Yi Chen, Po-Lin Kuo

**Affiliations:** ^1^Division of Pulmonary and Critical Care Medicine, Kaohsiung Medical University Hospital, Kaohsiung 807, Taiwan; ^2^Department of Internal Medicine, Kaohsiung Municipal Ta-Tung Hospital, Kaohsiung 801, Taiwan; ^3^Graduate Institute of Medicine, Kaohsiung Medical University, Kaohsiung 807, Taiwan; ^4^Department of Beauty Science, National Taichung University of Science and Technology, Taichung 403, Taiwan; ^5^School of Medical and Health Sciences, Fooyin University, Kaohsiung 831, Taiwan; ^6^Institute of Clinical Medicine, College of Medicine, Kaohsiung Medical University, Kaohsiung 807, Taiwan; ^7^Cancer Center, Kaohsiung Medical University Hospital, Kaohsiung 807, Taiwan; ^8^Department of Medical Research, Kaohsiung Medical University Hospital, Kaohsiung 807, Taiwan

## Abstract

This study investigated the anticancer effects of subamolide A (Sub-A), isolated from *Cinnamomum subavenium*, on human nonsmall cell lung cancer cell lines A549 and NCI-H460. Treatment of cancer cells with Sub-A resulted in decreased cell viability of both lung cancer cell lines. Sub-A induced lung cancer cell death by triggering mitotic catastrophe with apoptosis. It triggered oxidant stress, indicated by increased cellular reactive oxygen species (ROS) production and decreased glutathione level. The elevated ROS triggered the activation of ataxia-telangiectasia mutation (ATM), which further enhanced the ATF3 upregulation and subsequently enhanced p53 function by phosphorylation at Serine 15 and Serine 392. The antioxidant, EUK8, significantly decreased mitotic catastrophe by inhibiting ATM activation, ATF3 expression, and p53 phosphorylation. The reduction of ATM and ATF3 expression by shRNA decreased Sub-A-mediated p53 phosphorylation and mitotic catastrophe. Sub-A also caused a dramatic 70% reduction in tumor size in an animal model. Taken together, cell death of lung cancer cells in response to Sub-A is dependent on ROS generation, which triggers mitotic catastrophe followed by apoptosis. Therefore, Sub-A may be a novel anticancer agent for the treatment of nonsmall cell lung cancer.

## 1. Introduction

Lung cancer has been one of the most common causes of death throughout the world for several decades [1, 2]. Despite significant advances in treatment, improving the therapeutic outcome remains a serious problem due to the high molecular variety and drug resistance [3]. Mitotic catastrophe is defined as a type of cell death caused by aberrant mitosis [4, 5]. In mammalian cells, mitotic catastrophe is defined as an aberrant form of mitosis associated with concurrent multinucleated giant cells that are temporarily viable but lose the ability to proliferate and eventually die by apoptosis or necrosis [6, 7]. Mitotic catastrophe can be induced by chemical stress, DNA damage, and chemotherapeutic agents like paclitaxel, doxorubicin, and nocodazole. Deficiencies in DNA damage response and cell cycle checkpoints of cancer cells make them more susceptible to the trigger of mitotic catastrophe by anticancer drugs [8–10]. Novel strategies targeting the activation of mitotic catastrophe is being developed in preclinical and clinical studies [11].

Reactive oxygen species (ROS) are produced by all aerobic cells to regulate cell development, growth, survival, and death, and drug metabolism [12–14]. Oxidative stress occurs when this critical balance is disrupted because of excess ROS production and/or antioxidant depletion [15, 16]. Evidence shows that many chemotherapeutic drugs may be selectively toxic to cancer cells by increasing oxidant stress and enhancing the already stressed cells beyond their limit [17, 18]. Cytotoxic ROS signaling appears to be triggered by cellular stress-related signaling, such as the ataxia telangiectasia-mutation (ATM) system, with consequent cell death [19, 20].

*Cinnamomum subavenium Miq.,* a tall evergreen tree belonging to the family Lauraceae, is mainly distributed in central and southern China, Burma, Cambodia, Taiwan, Malaysia, and Indonesia [21, 22]. Recent studies reveal that it can act as a melanogenesis inhibitor by targeting tyrosinase, thereby providing safer and more effective ways of skin lightening [23]. One of the extracted compounds, Subamolide A ((3Z,4R,5R)-3-tetradecylidene-4-hydroxy-5-methoxy-5-methylbutanolide; Sub-A), is reported to have anticancer activity in human urothelial carcinoma NTUB1 cells [23]. However, there has been no other study on sub-A and its antilung cancer activities and mechanisms. This study focused on sub-A, its antilung cancer activity, and the possible mechanisms for the potential of developing new anticancer drugs.

## 2. Materials and Methods

### 2.1. Cell Culture

Human lung cancer cell lines A549 and NCI-H460, and normal lung fibroblast IMR-90 (ATCC CCL-186) were incubated at 37°C in a 5%  CO_2_-containing incubator. Minimum essential medium (MEM), with 10% FBS, 100 units/mL penicillin G, 100 *μ*g/mL streptomycin, 0.25 *μ*g/mL Amphotericin B, nonessential amino acids, and 0.1 mM sodium pyruvate, was used. The medium was changed once every 2-3 days and the cells were channeled after distinct cell density. 

### 2.2. Subamolide A (Sub-A) Preparation

**Figure 1:**
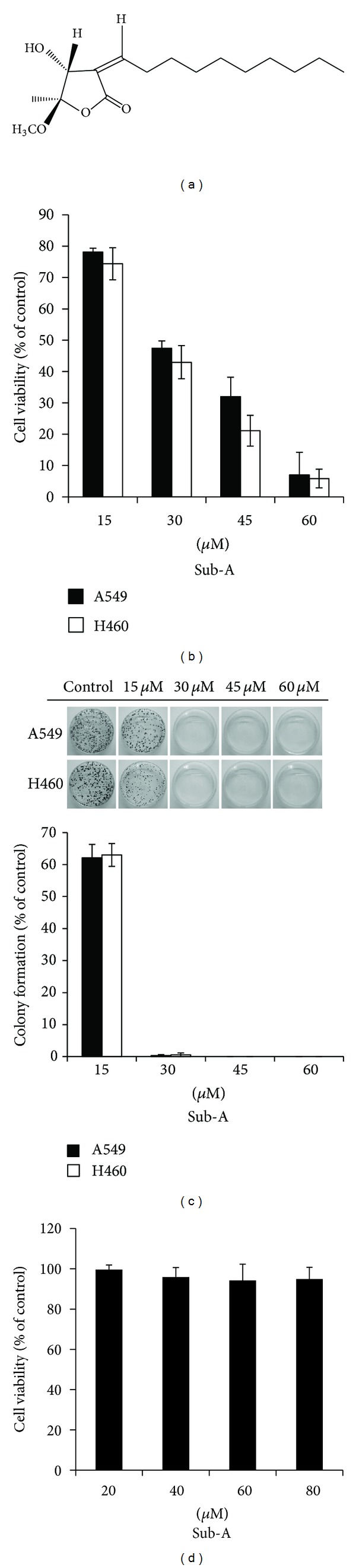
The effects of Sub-A on cell proliferation and colony formation in lung cancer cell lines A549 and NCI-H460. (a) The chemical structure of Sub-A isolated from the leaves of *Cinnamomum subavenium Miq.* (b) Decreased A549 and NCI-H460 cell viability by Sub-A. (c) The influence of A549 and NCI-H460 on the number of colony-forming cells, as evaluated by clonogenic assay. (d) Sub-A did not affect the cell viability in IMR-90 cells. The cell viability was assessed by trypan blue staining after 48 h treatment at difference of concentration of Sub-A. For colony-forming assay, the clonogenic assay was performed as described in Section 2. Results were expressed as the percentage of cell proliferation relative to the proliferation of the control. The data shown are the mean from three independent experiments. Each value in the data was the mean ± SD of three determinations.

Subamolide A (purity >90%) (Figure 1(a)) was isolated from the stems of *Cinnamomum subavenium* as described previously [22, 23]. Briefly, the air-dried stems were extracted with MeOH at room temperature. The MeOH extract, obtained by concentration under reduced pressure, was suspended in H_2_O and then partitioned with CHCl_3_ to yield fractions soluble in CHCl_3_ and H_2_O. The CHCl_3_-soluble fraction was chromatographed over silica gel using *n*-hexane-EtOAc-MeOH mixtures as eluents and separated into five fractions. Fraction 2 was resubjected to silica gel column chromatography and purified by preparative thin-layer chromatography using *n*-hexane-EtOAc to yield subamolide A. 

### 2.3. Cell Proliferation and Colony Formation Assay

Trypan blue exclusion test was used to determine the number of viable cells in a cell suspension. Cells (1 × 10^4^/well) were seeded in a 12-well plate and incubated in 37°C for 24 h. After the cells attached to the plate, different concentrations of Sub-A were added and the cells were incubated for another 48 h. They were then removed and mixed with 10 *μ*L 0.4% trypan blue solution. Unstained (viable) and stained (nonviable) cells were counted. 

To determine the long-term effects, cells (1 × 10^3^) were treated with different concentrations of Sub-A for 6 h. The medium was changed every 2-3 days and the cells were allowed to grow for 14 days. At the end, the cells were rinsed with PBS, fixed with 4% formaldehyde for 30 min in room temperature, and stained with 0.4 g/L crystal violet. The number of cell colonies was then recorded.

### 2.4. Apoptosis Analysis

The cells (1.5 × 10^6^) were treated with different concentrations of Sub-A for 48 h. DNA was extracted and separated by 2% agarose gel electrophoresis. The gel was stained with 0.5 mg/mL ethidium bromide (EtBr), placed on a UV transilluminator, and then photographed.

Quantitative analysis was conducted by the terminal deoxynucleotidyl transferase-mediated deoxyuridine triphosphate nick end-labeling (TUNEL) method using the BD ApoAlert DNA Fragmentation Assay Kit. The stained cells were analyzed by flow cytometer (Becton Dickinson and Co.) and fluorescence microscope (Nikon Eclipse TE 300, Germany) equipped with a filter system (emission filter 520 nm) at 20x magnification.

### 2.5. Immunofluorescence

The cells (6.5 × 10^4^) were cultured on glass chamber slides and treated with different concentrations of Sub-A and vehicle (0.1% DMSO) for 24 h after the cells were attached. The cells were fixed by 4% paraformaldehyde and incubated with 1% bovine serum albumin in phosphate buffered saline containing 0.1% Tween-20 (PBST). They were then incubated in the mixture of primary antibodies overnight at 4°C. After washing with PBST, the slides were incubated with Dylight 488 or Dylight 549-conjugated secondary antibodies (Rockland, Gilbertsville, PA), with or without DAPI, for 1 h at room temperature. Data were analyzed using a confocal laser scanning microscope (Fluoview FV1000, Olympus, Tokyo, Japan).

### 2.6. Measurement of ROS and Glutathione

Intracellular ROS and glutathione (GSH) levels were measured using H_2_DCFDA and CMFDA, respectively. Briefly, the cells were washed with PBS and incubated with 10 *μ*M H_2_DCFDA and 10 *μ*M CMFDA for 30 min. The fluorescence intensities were measured using an EPICS profile flow cytometer.

### 2.7. Quantitative Real-Time Polymerase Chain Reaction (qRT-PCR)

RNA isolation was performed using the TRIzol reagent (Invitrogen). cDNA was prepared using an oligo (dT) primer and reverse transcriptase (Takara, Shiga, Japan) following standard protocols. qRT-PCR was performed using SYBR Green on the ABI 7500 Real-Time PCR System (Applied Biosystems, Foster City, CA, USA). Each PCR reaction mixture contained 200 nM each primer, 10 *μ*L 2x SYBR Green PCR Master Mix (Applied Biosystems, Foster City, CA), 5 *μ*L cDNA, and RNase-free water for a total volume of 20 *μ*L. The PCR reaction was performed with a denaturation step at 95°C for 10 min, and then for 40 cycles at 95°C for 15s and 60°C for 1 min. All PCRs were performed in triplicate and normalized to the internal control glyceraldehyde-3-phosphate dehydrogenase (GAPDH) mRNA. Relative expression was presented using the 2^−ΔΔCT^ method. 

### 2.8. Immunoblot

The cells were treated with 30 *μ*M Sub-A and M-PER lysis buffer. The cell lysate was centrifuged at 14,000 ×g for 15 min, and the supernatant fraction was collected for immunoblot. Equivalent amounts of protein were resolved by SDS-PAGE (6–12%) and transferred to PVDF membranes. After blocking for 1 h in 5% nonfat dry milk in Tris-buffered saline, the membrane was incubated with the desired primary antibody for 1–16 h. The membrane was then treated with appropriate peroxidase-conjugated secondary antibody and immunoreactive proteins were detected using an enhanced chemiluminescence kit (Amersham, USA) according to the manufacturer's instructions. 

### 2.9. Gene Knockdown

Knockdown of ATM and ATF3 in A549 and NCI-H460 cells was performed using a lentiviral expression system provided by the National RNAi Core Facility (Taipei, Taiwan). Lentiviruses were produced by cotransfecting HEK293T with pLKO-AS2, pLKO-AS2-ATM or pLKO-AS2-ATF3 shRNA, and two packaging plasmids (pCMVVDR8.91 and pMD.G). Stable clones were established by puromycin selection. 

For p53 inhibition, the cells were exposed to the mixture of Lipofectamine 2000 reagent and pCMV-p53mt135 plasmid or empty vector for 6 h. After transfection, cells resistant to neomycin were selected by incubating with medium containing 1 mg/mL G418 (geneticin) (Life Technologies). Individual clones were isolated and tested for constitutive p53 expression. The p53mt135-positive and -negative (control) cells were selected and maintained in the presence of G418 (400 *μ*g/mL).

### 2.10. TEM (Transmission Electron Microscope)

Tumor sections were directly fixed with 2% paraformaldehyde and glutaraldehyde, postfixed with 2% osmium tetroxide, and embedded in epoxy resin. Representative areas were chosen for ultrathin sectioning and viewed with a JEM 1010 transmission electron microscope (JEOL USA INC. Peabody, MA).

### 2.11. *In Vivo* Tumor Xenograft Study

Male nude mice (4 weeks old; *n* = 21) were purchased from the National Laboratory Animal Center (Taipei, Taiwan) and maintained in pathogen-free conditions. A549 cells (2 × 10^7^ cells/200 *μ*L PBS) were injected subcutaneously into the mice and the tumors were allowed to develop to approximately 100 mm^3^. The mice were then randomly divided into three groups as follows: (1) 4% Cremophor EL per day (intraperitoneal injection; IP) five times a week for 53 days; (2) 10 mg/kg/day Sub-A dissolved in 4% Cremophor EL five times a week for 53 days; and (3) 4 mg/kg/day Sub-A dissolved in 4% Cremophor EL five times a week for 53 days. The mice were treated with equal volumes of vehicle.

After transplantation, tumor size was measured using calipers, while the tumor volume was estimated using the formula:

(1) $$\begin{array}{c} \text{Tumor}\;\text{volume}\left({\text{mm}}^{3}\right)=\frac{\text{long}\;\text{diameter}\times \text{short}\;{\text{diameter}}^{2}}{2}. \end{array}$$

Tumor-bearing mice were sacrificed 53 days after dosing. Sections of the tumors were used to determine apoptosis by DNA Fragmentation and TEM assay.

### 2.12. Statistical Analysis

All data results were expressed as Mean ± SD and analyzed by one-way analysis of variance (ANOVA). Differences between the experimental and control groups were analyzed using the Student's *t*-test. A *P* < 0.05 was considered statistically significant.

## 3. Results

### 3.1. Sub-A Inhibited the Proliferation of Human Lung Cancer Cell Lines A549 and NCI-H460

To investigate the potential cytotoxic activity of Sub-A in human lung cancer, we first examined the effect of Sub-A on cell viability and colony formation in both A549 and NCI-H460 cell lines. Exposure of the human cancer cell lines A549 and NCI-H460 to Sub-A for 48 h decreased the cell viability of each cell line in a dose-dependent manner (Figure 1(b)). The anticancer activities of Sub-A were also assessed by clonogenic assays (Figure 1(c)). Compared to the Sub-A-treated group, both A549 and NCI-H460 cell lines were able to form clones in the vehicle-treated group. When Sub-A was added, there was a dose-dependent inhibition in clonogenicity, with 50% inhibition at dosages as low as 30 *μ*M (Figure 1(c)). To examine the selection of Sub-A cytotoxicity, we also evaluated the effect of Sub-A in normal fibroblast cell line, IMR-90. The results showed that treatment of IMR-90 cells with Sub-A failed to affect the cell viability (Figure 1(d)). This result demonstrated that Sub-A possessed selectivity between normal and cancer cells.

### 3.2. Sub-A Induced Mitotic Catastrophe in A549 and NCI-H460 Cells

**Figure 2:**
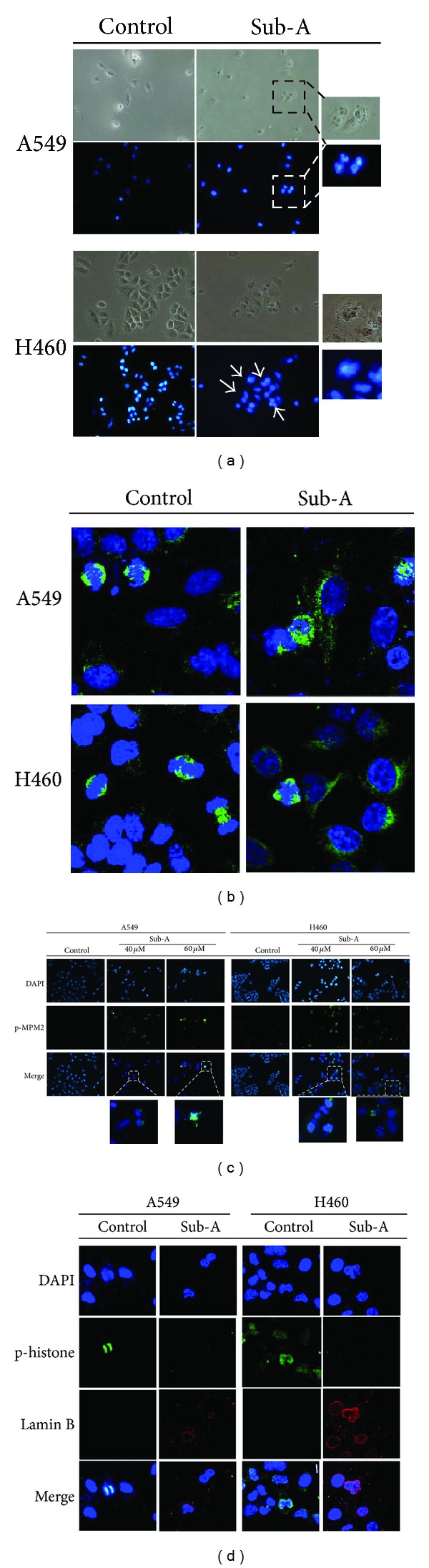
Sub-A induced mitotic catastrophe in A549 and NCI-H460 cells. (a) The formation of multinucleated giant cells in Sub-A-treated cells. (b) Aberrant segregation of chromosomes. (c) Accumulation of p-MPM2. (d) Abnormal mitosis. Cells were treated with 30 *μ*M Sub-A for 24 h and then were stained by DAPI, tubulin antibodies, p-MPM2 antibodies, or p-histone3/Lamin B antibodies. The samples were analyzed by confocal microscopy and the data were representative of three independent experiments.

We found that cells displayed a mitotic catastrophe phenotype, enlarged micro- or multinucleated cells, after 24 h treatment. Therefore, we further investigated if Sub-A caused mitotic catastrophe in lung cancer cells. The formations of multinuclear formations of multinuclear giant cells were observed in Sub-A-treated A549 and NCI-H460 cells (Figure 2(a)). Staining for *α*-tubulin also showed that Sub-A treatment caused aberrant segregation of chromosomes, microtubule misalignment, multipolar mitosis, and MPM-2-positive multi-nucleated cells in both cancer cell lines (Figures 2(b) and 2(c)). 

Counterstaining phosphorylated Histone 3 (H3) Ser10 and Lamin B was another method to define mitotic catastrophe. Normal mitotic cells were positive for phosphorylated H3 (at Ser10) stains but negative for lamin B1 staining due to the loss of integrity of the nuclear envelope during mitosis. In contrast, cells undergoing mitotic catastrophe were negative for phosphorylated H3 staining but positive for lamin B staining due to failed cytokinesis. With the comparison to vehicle treatment, the exposure of A549 and NCI-H460 cells with Sub-A caused mitotic catastrophe (Figure 2(d)). 

### 3.3. Sub-A Induced Apoptosis in A549 and NCI-H460 Cells

**Figure 3:**
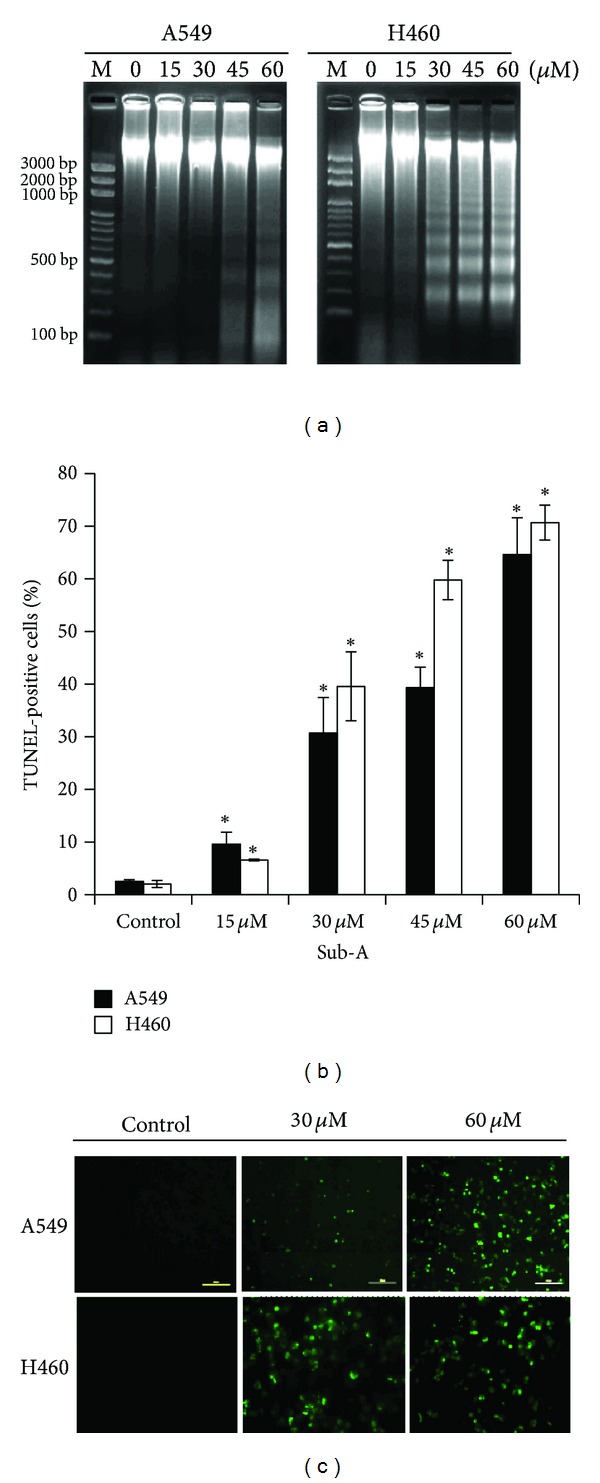
Sub-A induced apoptosis in A549 and NCI-H460 cells. (a) The DNA fragmentation of A549 and NCI-H460 cells induced by Sub-A, as determined by electrophoresis assay at 48 h. Quantitative evaluations of TUNEL assay by (b) flow cytometry and (c) fluorescence microscopy. Each value was the mean ± SD of three determinations. The asterisk indicated a significant difference between Sub-A-treated cells and the controls (*P* < 0.05, by ANOVA with Student's *t*-test post hoc).

Under light microscope we observed a substantial fraction of shrinking cells after 48 h treatment. Sub-A treatment resulted in the formation of DNA fragments in both cancer cell lines, shown by agarose gel electrophoresis at the indicated times (Figure 3(a)). Compared to vehicle-treated cells, Sub-A-treated A549 and NCI-H460 cells showed apoptosis after 48 h (Figure 3(b)). TUNEL-positive cells were also visible by fluorescence microscopy (Figure 3(c)).

### 3.4. Sub-A Raised Cytosol ROS Level/ATM System

**Figure 4:**
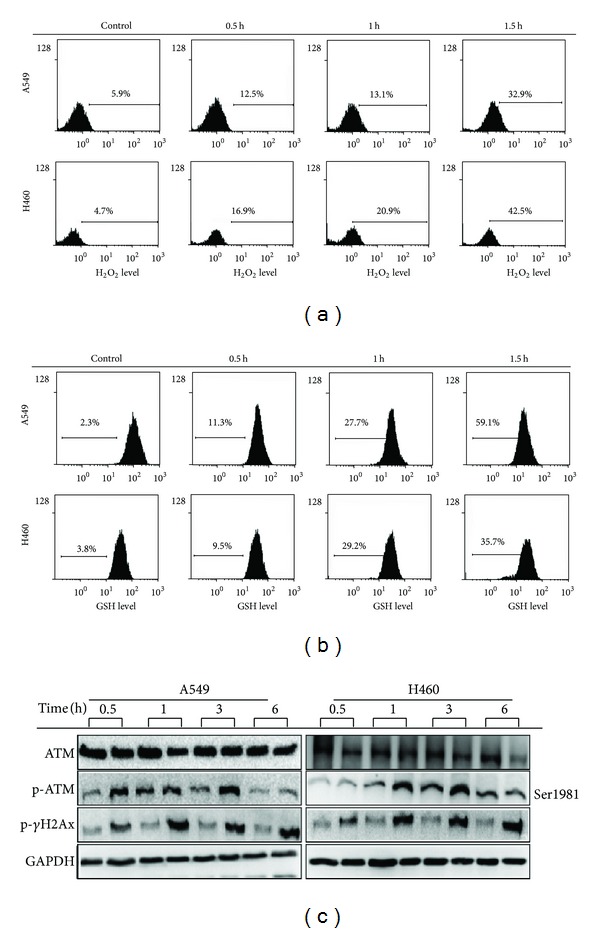
The effect of Sub-A on the production of ROS, glutathione levels, and ATM activation. (a) Sub-A increased the generation of ROS in the A549 and NCI-H460 cells. (b) Sub-A decreased the glutathione levels but (c) increased ATM activation in the A549 and NCI-H460 cells. Cells were treated with 30 *μ*M Sub-A for the indicated times, and the amounts of ROS and glutathione were assayed by H_2_DCFDA (for ROS) and CMFDA (for glutathione) staining, while the activation of ATM was assessed by immunoblot assay.

Because ROS generation plays important roles in the regulation of cell fates [17, 18], we then investigated whether Sub-A altered the generation of ROS in lung cancer cells. Sub-A treatment increased the mean DCF fluorescence in both A549 and NCI-H460 lung cancer cells (Figure 4(a)). Glutathione (GSH) scavenges ROS in cells by interacting with OH^∙^ and H_2_O_2_, thus affecting ROS-mediated signaling pathways. We next examined the levels of GSH in Sub-A-treated cells. Treatment of Sub-A also effectively decreased GSH levels in both lung cancer cell lines. These data suggested that ROS might play a role in Sub-A-mediated cell death (Figure 4(b)).

There is growing evidence that ROS-mediated DNA damage triggers the activation of ATM system [19, 20]. Therefore, we assessed the status of ATM after Sub-A treatment in lung cancer cells. Sub-A increased the phosphorylation (Ser1981) of ATM protein in both A549 and NCI-H460 cells but did not cause any change in the protein levels of total ATM (Figure 4(c)). Exposure of A549 and NCI-H460 cells to Sub-A also resulted in increased levels of the phosphorylated (activated) form of H2A.X (Ser139), a variant form of histone H2A that was directly phosphorylated at Ser139 by activated ATM kinase. These findings suggest that Sub-A triggers the ROS/ATM system in lung cancer cells. 

### 3.5. Sub-A Increased the Expression of ATF3 and p53

**Figure 5:**
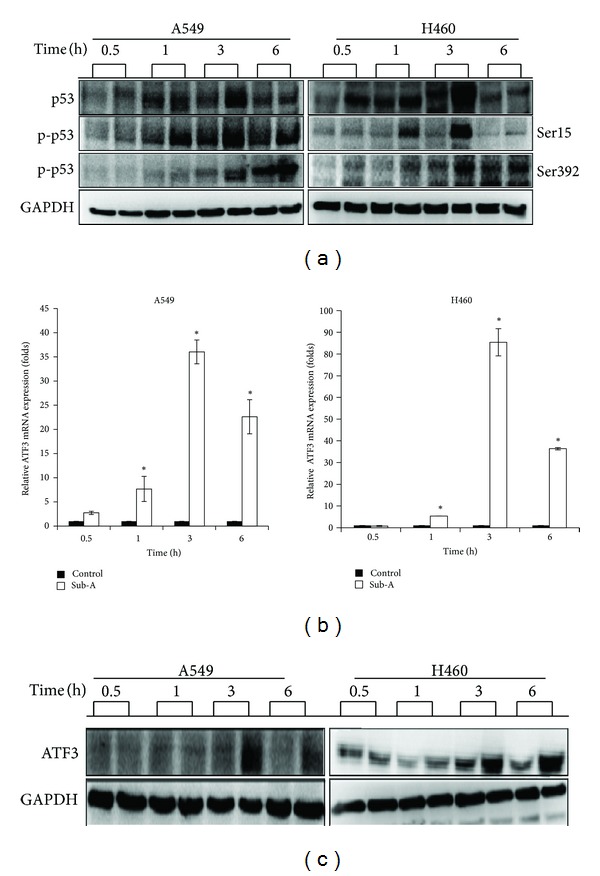
The effect of Sub-A on p53 regulation and ATF3 expression. (a) Sub-A increased the level and phosphorylation of p53. Sub-A increased the ATF3 expression at (b) mRNA and (c) protein levels. Cells were treated with 30 *μ*M Sub-A for the indicated times, and the amounts of mRNA and protein were assessed by qRT-PCR and immunoblot assay, respectively. Each value was the mean ± SD of three determinations. The asterisk indicated a significant difference between Sub-A-treated cells and the controls (*P* < 0.05, by ANOVA with Student's *t*-test post hoc).

Since p53 was described to be one of major players in determination of cell fate [24], we assessed the status of p53 in Sub-A-treated A549 and NCI-H460 cells. Exposure to Sub-A not only increased p53 expression but also enhanced p53 phosphorylation on Ser15 and Ser392 after 3 h in A549 and NCI-H460 cells (Figure 5(a)).

Previous study showed that ROS stress can enhance activating transcription factor 3 (ATF3) expressions that can modulate the activity of p53 [25, 26]. We assessed the expression of ATF3 at both mRNA and protein levels. Sub-A for 1 h also enhanced the expression of ATF3 in both mRNA and protein levels of A549 and NCI-H460 cells (Figures 5(b) and 5(c)). 

### 3.6. The Roles of ROS and ATM in Sub-A-Mediated Mitotic Catastrophe

**Figure 6:**
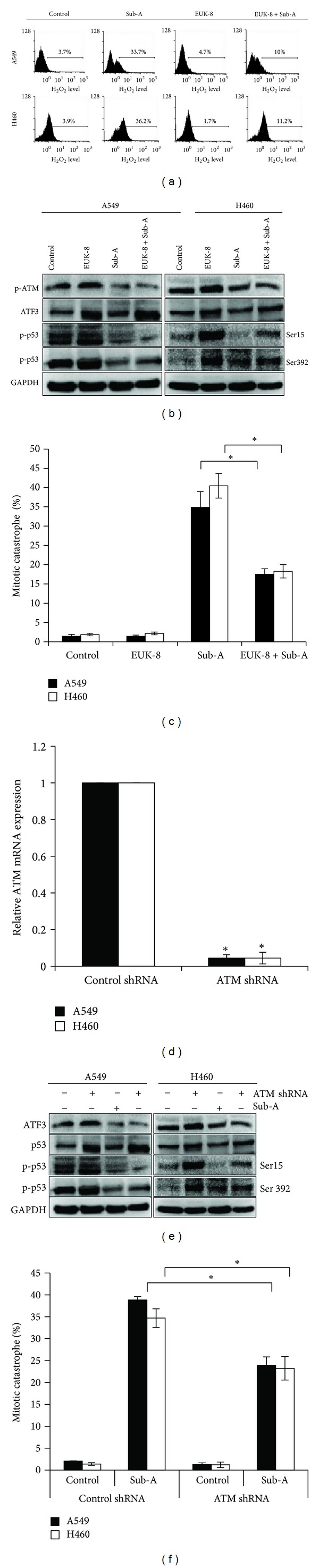
The role of ROS and ATM on Sub-A-mediated mitotic catastrophe. Antioxidant agents inhibited (a) Sub-A-mediated ROS generation, (b) ATM activation and ATF3 upregulation, and (c) mitotic catastrophe. (d) The effect of ATM shRNA transfection on the expression of ATM. Inhibition of ATM decreased the (e) phosphorylation of p53 and (f) mitotic catastrophe. For (a) to (c), cells were pretreated with EUK8 (50 mmol/L) for 1 h, then 30 *μ*M Sub-A was added and incubated for specific times (1 h for ATM activity and 24 h for mitotic catastrophe). For (d) to (f), cells were transfected control or ATM shRNA and then incubated with 30 *μ*M Sub-A for specific times (3 h for p53 phosphorylation and 24 h for mitotic catastrophe). The induction of mitotic catastrophe was determined by counting multinuclear giant cells after DAPI staining. Each value was the mean ± SD of three determinations. The asterisk indicated a significant difference between Sub-A-treated cells and the controls (*P* < 0.05, by ANOVA with Student's *t*-test post hoc).

To establish the sequential occurrence between ROS, ATM activation, ATF3 expression, and p53 expression, ROS was scavenged by EUK-8, a superoxide dismutase and catalase mimetic. EUK-8 (50 *μ*M) pretreatment decreased Sub-A-induced ROS generation (Figure 6(a)). Pretreatment with EUK-8 not only inhibited Sub-A-induced ATM activation, ATF3 expression, and p53 phosphorylation (Figure 6(b)), but also blocked Sub-A-induced mitotic catastrophe (Figure 6(c)). Thus, ROS might be involved Sub-A-induced ATM activation, ATF3 expression, in p53 phosphorylation, and in mitotic catastrophe.

To determine if any of the known ATM plays a role in Sub-A-mediated mitotic catastrophe, we silenced ATM by specific shRNA. ATM shRNA knockdown of ATM markedly inhibited its mRNA expression in both cancer cell lines (Figure 6(d)). Selective genetic inhibition of ATM also abrogated ATF upregulation and p53 phosphorylation (Figure 6(e)). Moreover, shRNA knockdown of ATM markedly reduced Sub-A-mediated mitotic catastrophe compared to those transfected with the control plasmid (Figure 6(f)). 

### 3.7. The Roles of ATF3 and p53 in Sub-A-Mediated Mitotic Catastrophe

**Figure 7:**
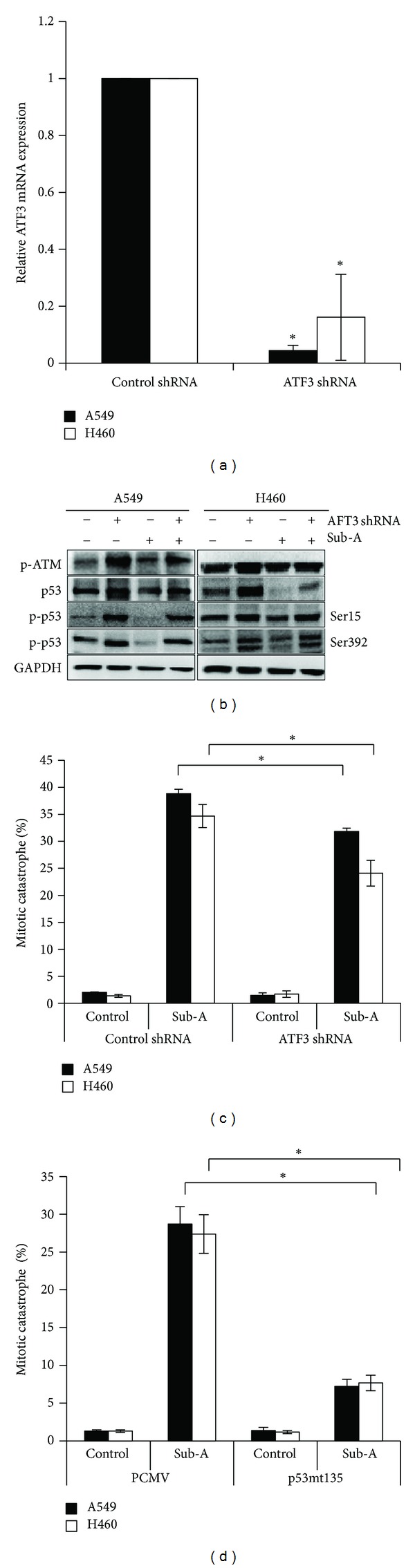
The role of ATF3 and p53 on Sub-A-mediated mitotic catastrophe. The inhibition of ATF3 decreased (a) ATF3 mRNA and (b) p53 levels in Sub-A-treated cells. (c) Knockdown of ATF3 reduced the Sub-A-mediated mitotic catastrophe. (d) The inhibition of p53 decreased the Sub-A-mediated mitotic catastrophe. Cells were transfected control or ATF3 shRNA and then incubated with 30 *μ*M Sub-A for specific times (3 h for p53 and 24 h for mitotic catastrophe). For (d), cells were transfected pCMV or pCMVp53mt135 plasmid, and then incubated with 30 *μ*M Sub-A for 24 h. The induction of mitotic catastrophe was determined by counting multinuclear giant cells after DAPI staining. Each value was the mean ± SD of three determinations. The asterisk indicated a significant difference between Sub-A-treated cells and the controls (*P* < 0.05, by ANOVA with Student's *t*-test post hoc).

To analyze the roles of ATF3 on Sub-A-mediated p53 expression and mitotic catastrophe, we examined the inhibition of ATF-3 by shRNA on the p53 status and mitotic catastrophe induction. ATF3 shRNA transfection inhibited the expression of ATF3 in ATFs shRNA plasmid A549 and NCI-H460 cells (Figure 7(a)). ATF-3 shRNA significantly reduced the expression of p53, but did not affect p53 phosphorylation and the ATM system (Figure 7(b)). Knockdown of ATF-3 by shRNA decreased the Sub-A-induced mitotic catastrophe in A549 and NCI-H460 cells (Figure 7(c)), suggesting that ATF-3 was upstream of p53. In addition, we further investigated the mechanism that accounts for the actions of p53 in Sub-A-mediated mitotic catastrophe in lung cancer cells by using dominant-negative fashion. Dominant-negative mutation of p53 transfection markedly reduced the Sub-A-induced mitotic catastrophe compared to those transfected with the control pCMV plasmid (Figure 7(d)).

### 3.8. Sub-A Inhibited Tumor Growth in Nude Mice

**Figure 8:**
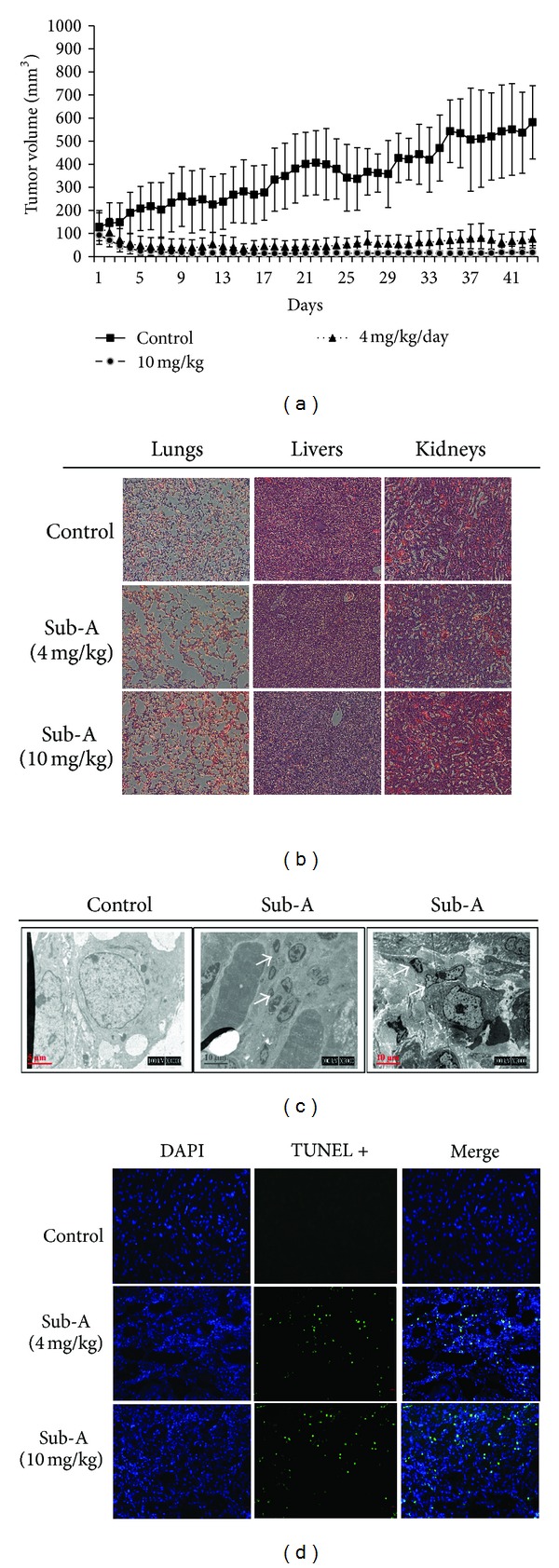
Sub-A inhibited growth of A549 in nude mice. (a) The mean of tumor volume measured at the indicated number of days after implantation. (b) There were no signs of toxicity, as judged by parallel monitoring of body weight and tissue sections of lungs, livers, and kidneys, in Sub-A-treated mice. Sub-A induced (c) mitotic catastrophe and (d) apoptotic cell death in the A549 xenograft. Animals bearing preestablished tumors (*n* = 12 per group) were dosed daily for 53 days with IP injections of low-dose Sub-A (4 mg/kg/d), high-dose sub-A (10 mg/kg/d), or vehicle. During the 53-day treatment, tumor volumes were estimated using measurements taken by external calipers (mm^3^). The induction of mitotic catastrophe was assessed by TEM and apoptosis was determined by TUNEL assay. The asterisk indicated a significant difference between Sub-A-treated cells and the controls (*P* < 0.05, by ANOVA with Student's *t*-test post hoc).

The xenograft model was used to assess whether Sub-A decreased the growth of A549 xenografts *in vivo*. The average tumor volume in mice treated with 4 mg/kg/day Sub-A was statistically significantly lower compared to vehicle-treated control mice (Figure 8(a)). Moreover, 10 mg/kg/day Sub-A completely inhibited the growth of A549 tumor in mice. Tissue sections of lungs, livers, and kidneys did not indicate any significant differences between vehicle- and Sub-A-treated mice (Figure 8(b)).

To gain insight into the mechanism of Sub-A inhibition of tumor growth *in vivo*, the A549 tumor xenografts were harvested from vehicle-treated and Sub-A-treated mice, and the apoptosis was assessed by TEM, TUNEL and immunoblot assay. The TEM analysis revealed multi-nucleated cells in the tumor section of Sub-A treated mice (Figure 8(c)). There was also an increase in TUNEL-positive cells in the tumors of Sub-A-treated mice, in contrast to cancer sections taken from vehicle-treated mice (Figure 8(d)). These findings suggest that Sub-A-mediated mitotic catastrophe and apoptosis in cultured cells and *in vivo* are correlated.

## 4. Discussion

Lung cancer is one of the most common cancers in developed and developing countries [1, 2, 27]. The major finding of the present study is that Sub-A, a natural compound isolated from *Cinnamomum subavenium*, effectively decreases human lung cancer cell viability via induction of mitotic catastrophe, which promotes apoptosis both *in vitro* and *in vivo*. The results also show that Sub-A treatment does not produce any overt signs of toxicity *in vivo*, thereby suggesting that Sub-A can discriminate between normal and cancer cells.

Mitotic catastrophe is a distinct form of programmed cell death that is widely used to describe a type of cell death occurring during mitosis [8–10, 28]. The induction of mitotic catastrophe is an attractive method for developing anticancer therapies due to several reasons. First, cancer cells are particularly sensitive to the induction of mitotic catastrophe due to their common tetraploidy/aneuploidy, which makes them more prone to mitotic aberrations. Second, unlike cell death induced by multiple chemotherapeutic agents at relatively high doses, mitotic catastrophe is induced at lower doses [29]. Third, inhibition of mitotic catastrophe is an important mechanism of chemoresistance in various cancers [30, 31]. In the present study, key features of mitotic catastrophe, including micronuclei and mitotic chromosomal missegregation, occur after mitosis and are apparent in Sub-A-treated lung cancer cells. Lastly, data from time-dependent studies indicate that the induction of mitotic catastrophe is followed by apoptosis, which in turn causes cell death. 

Enhancement of ROS production has long been associated with the therapeutic principle of several anticancer agents for selectively killing cancer cells [17, 18, 32]. The condition of intracellular redox reaction is modulated by antioxidant enzymes and nonenzymatic antioxidants [15, 16]. Glutathione (GSH) is a major thiol-disulphide redox buffer that participates in the maintenance of a reducing environment in the cell. The upregulation of GSH levels contributes to the protection of cancer against apoptosis. It is a critical factor for chemoresistance to various anticancer agents [33, 34]. Consequently, low GSH levels are associated with mitochondrial dysfunction and the induction of cell death, thereby increasing sensitivity to anticancer drugs [35]. 

In the present study, Sub-A treatment causes a significant accumulation of ROS that parallel a decrease in GSH. Even though the antioxidant, EUK8, effectively antagonizes Sub-A-mediated mitotic catastrophe, Sub-A treatment also induces a classic ROS-triggered stress response pathway involving ATM activation and ATF3 induction in both A549 and NCI-H460 cells. Moreover, although the induction of oxidative stress is not shown to be involved in the induction of mitotic catastrophe, ROS plays an important role in determining cell fate of either tumorigenesis or cell death. Given the findings, it thus seems plausible that the generation of ROS contributes to the selective antitumor activity of Sub-A in lung cancer.

The ATM pathway has been involved in triggering the cell cycle checkpoint or cell death by DNA damage-dependent and -independent means [36]. Once activated, ATM phosphorylates various downstream molecules, such as p53, H2A.X, and the Nijmegen breakage syndrome, resulting in cell cycle arrest or cell death [37]. ATM phosphorylates p53 at Ser15, resulting in prolonged p53 half-life by inhibiting the p53-MDM2 complex formation. ATM also phosphorylates p53 at Ser392 to enhance the transcriptional activity of p53 [38, 39]. p53 has been shown to play a critical role in the regulation of mitotic catastrophe resulting from cells entering mitosis with DNA damaged stress [37, 40]. 

The present study shows that treatment of A549 and NCI-H460 cells with Sub-A results in the accumulation of phospho-ATM at Ser1981. This ATM activation correlates well with the Sub-A-induced increase in H2A.X phosphorylation. Furthermore, the inhibition of ATM by shRNA decreases the Sub-A-induced ATM activation and prevents p53 phosphorylation (Ser15 and 392), suggesting that Sub-A-induced ATM activation contributes to the stabilization and enhancement of p53 function. The results also show that exposure of A549 and NCI-H460 cells to Sub-A leads to concurrent of mitotic catastrophe, whereas decreased ATM transfection inhibits Sub-A-mediated mitotic catastrophe. This implies that the activation of ATM induced by Sub-A is involved in the induction of mitotic catastrophe. Lastly, the inhibition of p53 via dominant-negative p53 also prevents Sub-A-induced mitotic catastrophe, further pointing to the cooperation of ATM and the p53-dependent pathway as a crucial feature of Sub-A-mediated mitotic catastrophe.

ATF3, a member of the ATF/cyclic AMP response element-binding (ATF/CREB) family of transcription factors, can be rapidly induced by a wide variety of signals, including those initiated by cytokines, genotoxic agents, or physiologic stresses [41–43]. ATF3 reportedly plays differing roles in the development of tumor, depending on the cell type and context. Elevated ATF3 expression has been reported in human breast cancers as protective against apoptosis or as promoting metastasis of cancer cells [44, 45]. In contrast, ATF-3 has been reported to play an important role in cisplatin-mediated apoptosis in lung cancer [46]. ATF3 stabilizes the p53 levels by decreasing ubiquitination and promotes degradation by disrupting the E6-E6AP interaction [47]. Hence, it is as an essential cotranscription factor for p53 on DNA damage. 

However, the mechanisms and transcriptional targets of ATF3 in tumor suppression remain largely undefined. The present study demonstrates that treatment of A549 and NCI-H460 cells with Sub-A results in enhanced ATF-3 whereas suppression of ATF3 via shRNA transfection decreases Sub-A-mediated p53 upregulation and mitotic catastrophe. ATF3 may therefore play an important role in the prolongation of p53 half-life and in cell death of Sub-A-treated lung cancer cells.

In conclusion, human lung cancer cells A549 and NCI-H460 are highly sensitive to Sub-A-induced mitotic catastrophe and apoptosis, mainly via ROS elevation that induces ATM and ATF3 activation, subsequently leading to p53-mediated cell death. Sub-A also causes cell growth inhibition in an *in vivo* xenograft model. The elucidated molecular bases and processes may provide a new strategy for developing more effective chemotherapeutic regimens for lung cancer treatment.
