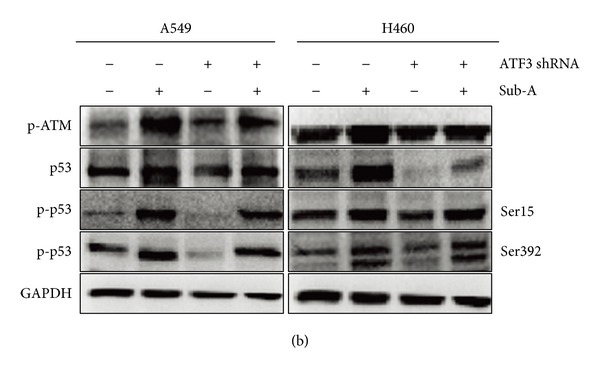# Erratum to “Subamolide A Induces Mitotic Catastrophe Accompanied by Apoptosis in Human Lung Cancer Cells”

**DOI:** 10.1155/2013/687142

**Published:** 2013-10-03

**Authors:** Jen-Yu Hung, Ching-Wen Wen, Ya-Ling Hsu, En-Shyh Lin, Ming-Shyan Huang, Chung-Yi Chen, Po-Lin Kuo

**Affiliations:** ^1^Division of Pulmonary and Critical Care Medicine, Kaohsiung Medical University Hospital, Kaohsiung 807, Taiwan; ^2^Department of Internal Medicine, Kaohsiung Municipal Ta-Tung Hospital, Kaohsiung 801, Taiwan; ^3^Graduate Institute of Medicine, Kaohsiung Medical University, Kaohsiung 807, Taiwan; ^4^Department of Beauty Science, National Taichung University of Science and Technology, Taichung 403, Taiwan; ^5^School of Medical and Health Sciences, Fooyin University, Kaohsiung 831, Taiwan; ^6^Institute of Clinical Medicine, College of Medicine, Kaohsiung Medical University, Kaohsiung 807, Taiwan; ^7^Cancer Center, Kaohsiung Medical University Hospital, Kaohsiung 807, Taiwan; ^8^Department of Medical Research, Kaohsiung Medical University Hospital, Kaohsiung 807, Taiwan

There are some errors that occurred during uploading Figures 5(a), 6(b), 6(e), and 7(b). The following are the corrected figures.

## Figures and Tables

**Figure 5:**
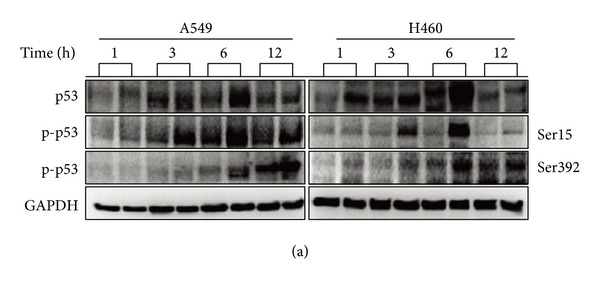


**Figure 6:**
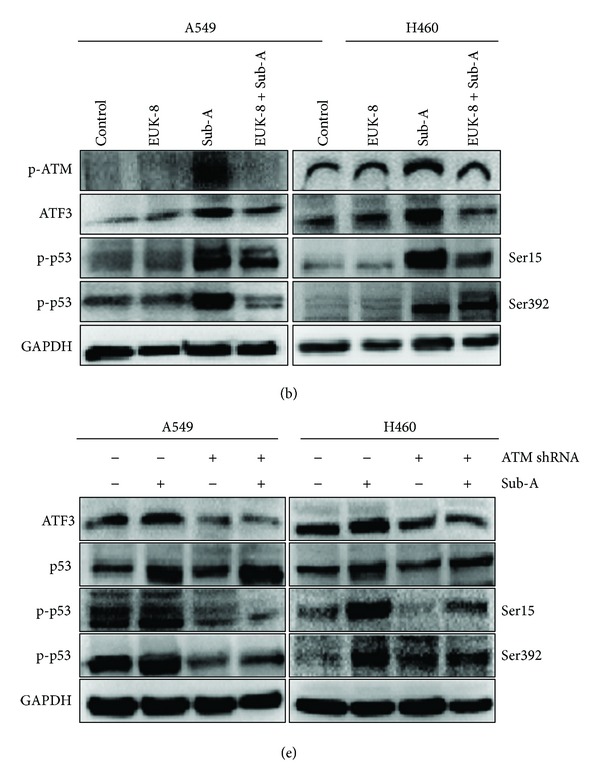


**Figure 7:**